# Identification of a circulating immunological signature predictive of response to immune checkpoint inhibitors in patients with advanced non‐small cell lung cancer

**DOI:** 10.1002/ctm2.1018

**Published:** 2022-08-22

**Authors:** Wassila Khatir, Olivier Humbert, Jonathan Benzaquen, Christophe Bontoux, Jaap Neels, Léa Berland, Fabian Andrés Gallardo Rivera, Maryline Allegra, Myriam Salah, Virginie Tanga, Olivier Bordone, Julien Fayada, Virginie Lespinet‐Fabre, Doriane Bohly, Elodie Long‐Mira, Sandra Lassalle, Valérie Vouret, Patrick Brest, Baharia Mograbi, Charlotte Maniel, Josiane Otto, Jacques Boutros, Simon Heeke, Véronique Hofman, Charles‐Hugo Marquette, Paul Hofman, Marius Ilié

**Affiliations:** ^1^ Laboratory of Clinical and Experimental Pathology Centre Hospitalier Universitaire de Nice FHU OncoAge Université Côte d'Azur Nice France; ^2^ Team 4, Institute of Research on Cancer and Aging (IRCAN), CNRS INSERM Centre Antoine‐Lacassagne Université Côte d'Azur Nice France; ^3^ Department of Nuclear Medicine Centre Antoine Lacassagne Université Côte d'Azur Nice France; ^4^ Department of Pulmonary Medicine and Thoracic Oncology Centre Hospitalier Universitaire de Nice FHU OncoAge Université Côte d'Azur Nice France; ^5^ Team Metabolic Challenges of Immune Cells in Obesity Diabetes, and Cardiovascular Disease, Centre Méditerranéen de Médecine Moléculaire (C3M), INSERM Université Côte d'Azur Nice France; ^6^ Department of Cancer Immunology and Virology Dana‐Farber Cancer Institute Boston Massachusetts USA; ^7^ Biobank Bellvitge Institute for Biomedical Research (IDIBELL) Barcelona Spain; ^8^ Biobank Côte d'Azur (BB‐0033‐00025) Centre Hospitalier Universitaire de Nice FHU OncoAge Université Côte d'Azur Nice France; ^9^ Department of Medical Oncology Centre Antoine Lacassagne Université Côte d'Azur Nice France; ^10^ Department of Thoracic H&N Medical Oncology UT MD Anderson Cancer Center Houston Texas USA

1

Dear Editor,

This pilot study suggests the presence of two different profiles of patients with advanced non‐small cell lung cancer (aNSCLC), responding to immune checkpoint inhibitors (ICIs). The first group has a functional T‐cell response with a favourable orientation towards an antitumour cytotoxic effect and few neutrophil‐associated cytokines. The second group has a weak functional T‐cell response, whereas their favourable response to ICIs may be related to the activation of the innate response.

Over the past decade, ICIs targeting PD‐1/PD‐L1 binding have brought considerable clinical benefits, with durable responses and increased OS in patients with aNSCLC.^1^ However, in clinical settings, the choice of treatment is based on a single‐tumour tissue biomarker (e.g. PD‐L1 expression in tumour cells), with ∼20% of patients responding favourably to ICIs.^2^ Thus, PD‐L1 assay provides punctual information on a single interaction within the immunological synapse, whereas the process of activating the immune response is much more complex and involves numerous other interactions. In addition to the recognition of antigens (Ag) and immune checkpoints, there is another level of activation of the adaptive response depending on the concentration of cytokines and other circulating immune molecules, activating various differentiation pathways and resulting in different phenotypes.^3^ Tissue biopsies can be used to examine the immune cell phenotype, in addition to a non‐invasive study of circulating immunological markers controlling immune cell recruitment and polarization. However, the circulating immunological signature does not directly reflect the inflammatory context of the tumour, and the concentrations at the periphery are lower, making challenging the measurement of their levels.^4^ We used TruCulture technology to stimulate T‐cells in vitro to assess the immunological signature and to investigate the T‐cell response, which can be damaged by a variety of factors. CD8+ chronic stimulation of T‐cells may cause them to become less functional, inhibiting an effective immune response.^5^ To determine a signature for identifying patients who respond favourably to ICIs, we analysed the expression of 30 circulating immune molecules in TruCulture and plasma samples collected prior to first‐line PD‐1/PD‐L1 inhibitors in patients with aNSCLC.

The heat map highlights a strong correlation between 10 cytokines, identifying two ‘clusters’ of patients, the ‘high concentrations’ group and the ‘low concentrations’ group (Figure 1A). The differences in concentration observed after stimulation of T‐cells were not related to differences in basal concentrations of the 10 cytokines analysed in matched plasma samples.

**FIGURE 1:**
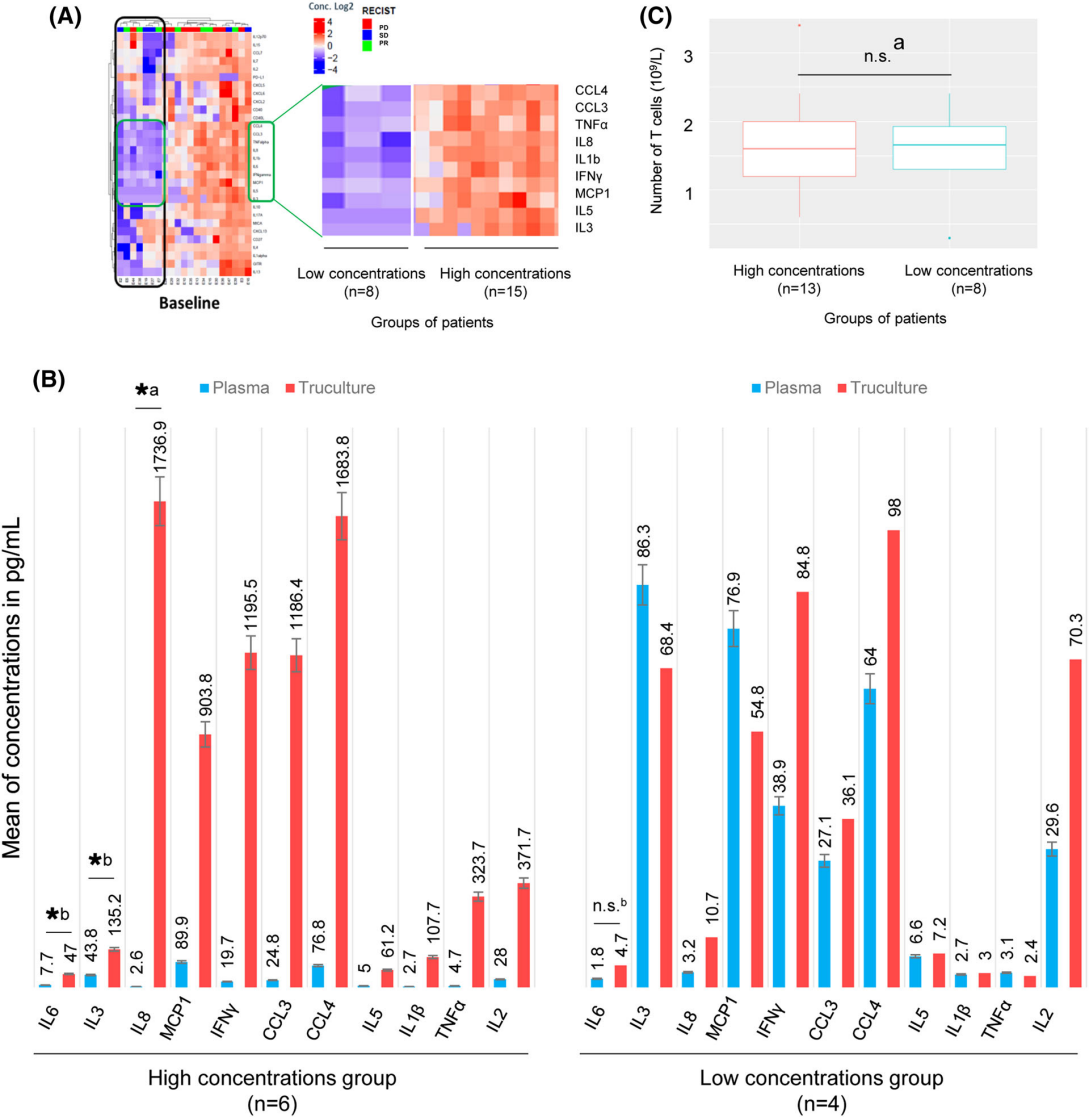
Identification of two profiles of patients differentiated by the functionality of their T‐cell response after TruCulture stimulation. (A) Heat map of the binary logarithm of the concentrations of 30 analytes (Luminex assay). Correlation of the concentrations was determined for each analyte. The correlation heat map is calculated in order to classify the patients and/or the analytes using a dendrogram gathering the patients/analytes having correlated profiles, the distance chosen being 1 − *R*, *R* being the correlation coefficient. Once this classification is calculated, the heat map presents an independent value of this correlation. Two groups of patients emerged. This grouping seems to depend on the concentration of 10 analytes. (B) Comparison of the levels of 10 analytes and of a marker of the activation of the T‐cell response, IL2, before and after stimulation with TruCulture for 10 patients from the two groups. Evidence of amplification of IL6, IL8, MCP1, IFNγ, CCL3, CCL4, IL5, TNFα and IL2 only in the group of patients with high concentrations. (C) Comparison of the number of T‐cells between the two groups of patients. No significant difference was observed. Significance code: .01 ‘*’; .05 ‘.’; .1 ‘n.s.’; 1: a, Welch test; b, Wilcoxon test

To investigate the hypothesis that the concentrations of these 10 markers reflect the level of activation of the T‐cell response, we compared their concentrations with those of IL2, a marker of T‐cell activation, in plasma (basal) and supernatant from TruCulture tubes (stimulated). In the group of patients with high concentrations, significant amplification of several analytes was observed (Figure 1B). No significant difference was observed in the low‐concentration group. These results suggest a difference in T‐cell activity in TruCulture (e.g. the ‘high concentrations’ group with a high T‐cell activity, and the ‘low concentrations’ group with low T‐cell activity). However, this difference in activity did not appear to affect the response to treatment, as responders were identified in either group. No difference in the number of T‐cells was observed between the two groups (Figure 1C), nor between responders and non‐responders (Figure 2A). This heterogeneity does not explain the responder/non‐responder status of the patients and could mask a signal of interest. We normalized the data with respect to the total marker concentration. Standardization allowed the percentage of each of the 30 targets represented within the patient profile to be defined, and the dominant signatures to be highlighted. To validate the relevance of standardization, the absence of an impact on the total concentration of patients' responses was demonstrated (Figure 2A). A significant correlation with the number of T‐cells was also observed (*p* = .0212). Moreover, the internal structure of the data was preserved because of normalization. The heat map highlighted the same groups of patients ‘low concentrations’ and ‘high concentrations’ as when analysing the non‐normalized results (Figure 2B). In the ‘high concentrations’ group, responders had a significantly lower percentage of CXCL5 (*p* = .028) and CXCL6 (*p* = .025) than non‐responders (Figure 3A). No significant difference was observed in the neutrophil/lymphocyte ratio of the patients (*p* = .1013), PD‐L1 expression (*p* = .093), *KRAS* mutations (*p* = .121) or TMB status (*p* = .097). The responders potentially had a profile enriched in TNFα and depleted in IL13. However, the *p*‐values were no longer significant after correction due to the small number of patients (Figure 3A,B).

**FIGURE 2:**
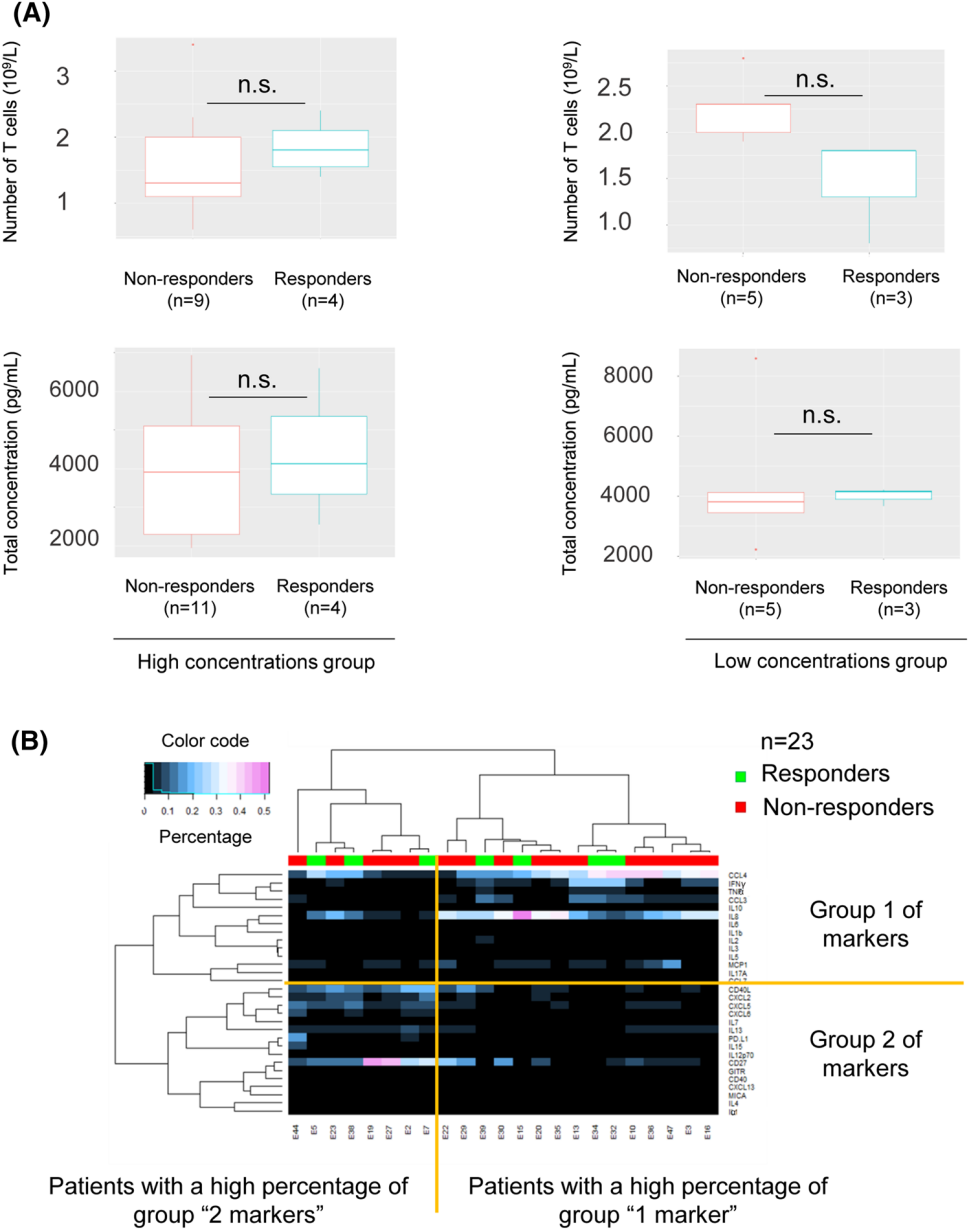
(A) Comparison of the number of T‐cells and total concentration (e.g. total concentration of the 30 targets) for responders and non‐responders in both groups. Significance code: .01 ‘*’; .05 ‘.’; .1 ‘n.s.’; 1, Wilcoxon test. (B) Study of the amplified immunological signature of 23 patients by multiplex analysis (Luminex). Heat map of correlation of normalized concentrations (%). The structure of the data is preserved, the clustering of patients and analytes are similar (dendrograms): two groups of patients associated with two different groups of targets. Group 1 contains the 10 markers previously studied in Figure 1.

**FIGURE 3:**
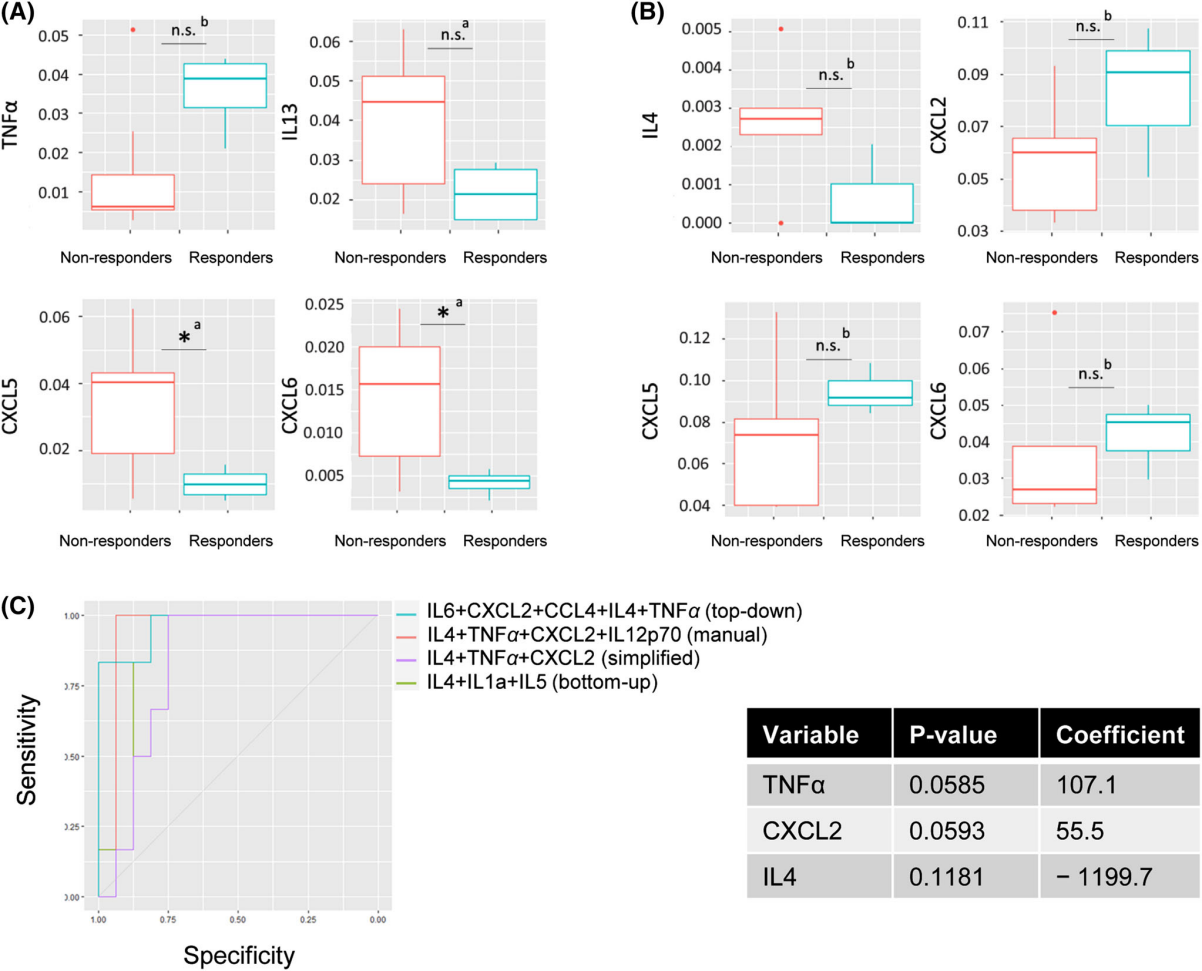
Amplified immunological signature analysis and identification of a predictive model for treatment response in patients with advanced non‐small cell lung cancer (NSCLC). (A) Comparison of analyte levels in responders and non‐responders in the group of patients with a functional T‐cell response. Only the four most relevant cytokines (lowest *p*‐values) are shown in this figure. Responders have significantly lower percentages of CXCL5 and CXCL6. They also have lower percentages of IL13 and higher percentages of TNFα. (B) Comparison of analyte levels in responders and non‐responders in the group of patients with a non‐functional T‐cell response. Only the four most relevant cytokines (lowest *p*‐values) are shown in this figure. No significant difference is observed before or after the Bonferroni correction. There is a slight trend. Responders potentially have higher percentages of CXCL2/5/6 and lower percentages of IL4. (C) (Left panel) Top‐down (AUC = .9532), manual (AUC = .9375) and bottom‐up (AUC = .8229) predictive model fitting. The best performing model had the highest AUC. It is both sensitive (detects many responders) and specific (detects few false responders). (Right panel) The combination of only three variables with the highest predictive power is TNFα + CXCL2 + IL4. High levels of IL4 are associated with an unfavourable response (negative coefficient), whereas high levels of TNFα and CXCL2 are associated with a favourable response (positive coefficient). Significance code: .01 ‘*’; .05 ‘.’; .1 ‘n.s.’; 1: a, Welch test; b, Wilcoxon test

In the ‘low concentrations’ group, responders seemed to have higher percentages of CXCL5 and CXCL6 and lower percentages of IL4 than non‐responders (Figure 3B). An inverse trend in CXCL5 and CXCL6 concentrations between the ‘high concentrations’ and ‘low concentrations’ low concentration groups was observed.

We next combined the variables to create a predictive model of responder status in both groups. High levels of IL4 were associated with an adverse response, whereas high levels of CXCL2 and TNFα were associated with a favourable response (Figure 3C).

To determine whether the response to ICIs was mediated by the activation of an antitumour innate response, we analysed the plasma levels of a target panel, including markers of polarization of the innate response. Responders appeared to have high levels of CXCL10 and low levels of CCL17 (Figure 4A). However, after correction, the *p*‐values were no longer significant. A trend was highlighted towards higher iNOS levels in responders (Figure 4B), suggesting that polarization of the innate response may play a role in the response to ICIs.

**FIGURE 4:**
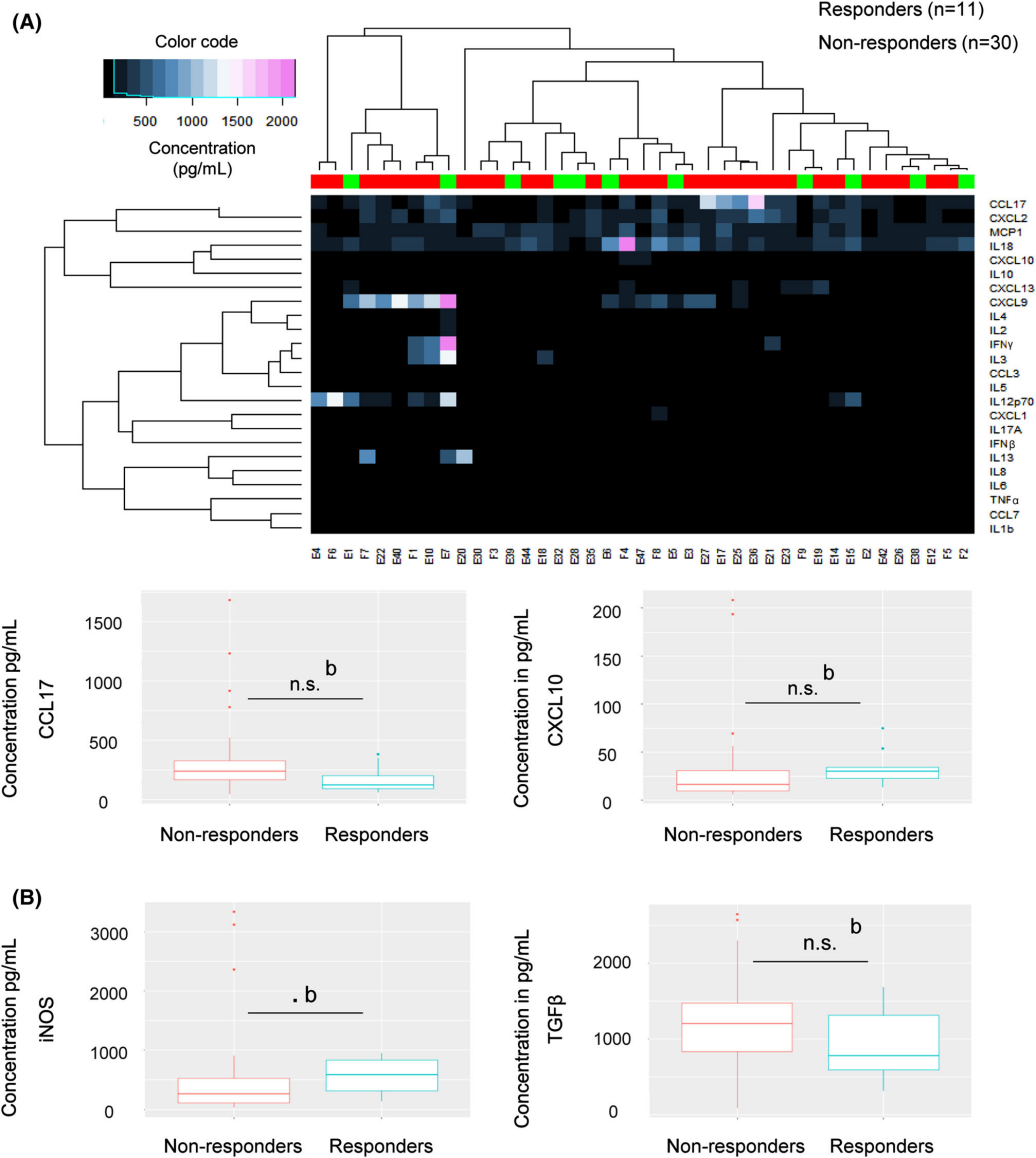
Analysis of the immunological signature in plasma of advanced non‐small cell lung cancer (NSCLC) patients and study of the orientation of the innate response. (A) Study of the immunological signature in plasma of 41 patients by Luminex analysis. Higher levels of CXCL10 and lower levels of CCL17 were observed in responders compared to non‐responders. Results not significant after the Bonferroni correction. (B) Comparison of plasma levels of TGFβ and iNOS (not exploitable in Luminex) in responders and non‐responders by ELISA assay. Demonstration of a trend: responders with higher levels of iNOS. Significance code: .01 ‘*’; .05 ‘.’; .1 ‘n.s.’; 1: a, Welch test; b, Wilcoxon test

In conclusion, our data suggest the existence of two populations of responders to ICIs, with either functional T‐cell response or non‐functional T‐cell response. We hypothesized that the polarization of the innate response could predict the responder/non‐responder status in both groups. This polarization may be in favour of a cytotoxic profile; the cytotoxic activity of the innate response could be taken over from the adaptive response in responders of the group ‘T‐cells with low functionality’. In addition, the polarization of the innate response may affect the polarization of the adaptive response towards a cytotoxic phenotype in the ‘functional T‐cells’ group.

## CONFLICT OF INTEREST

The authors report no conflict of interest.

## FUNDING INFORMATION

This work was funded by ‘Fondation ARC pour la Recherche sur le Cancer – Sign'it 2019’, ‘Fondation Bristol Myers Squibb pour la recherche en immuno‐oncologie’, IDEX Université Côte d'Azur, Cancéropôle PACA and ‘Conseil Départemental des Alpes‐Maritimes’.

## Supporting information


Supporting Information
