# Supplementary material for: Identification of a circulating immunological signature predictive of response to immune checkpoint inhibitors in patients with advanced non‐small cell lung cancer
Source: Clin Transl Med. 2022 Aug 22;12(8):e1018. doi: 10.1002/ctm2.1018 (PMC9394752; doi:10.1002/ctm2.1018)
Supplement: Supplementary file 1 — Supporting Information [file CTM2-12-e1018-s001.docx]

**SUPPORTING MATERIAL**

**MATERIALS AND METHODS**

**Patients**

The screened study population consisted of 47 patients treated with anti-PD-1/PD-L1 inhibitors for stage IIIB–IV NSCLC at the Antoine Lacassagne Comprehensive Cancer Center (Nice, France). Participants were prospectively recruited for blood sampling between September 2018 and May 2019. The inclusion criteria were as follows: patients older than 18 years, written consent to the collection and storage of blood samples, availability of clinical samples during the study window, measurable tumor lesions to be able to define the patient's response, and no influence of the patient's state of health prior to treatment (e.g., ECOG performance status between 0 and 2). In all, 23 patients were included in the analysis after applying inclusion criteria.

The clinical response to anti-PD-1/PD-L1 therapy was determined as the best response based on immune-related RECIST (iRECIST) [[1](#_ENREF_1)] at the 12-week time point and classified as responders [e.g., complete response (CR) and partial response (PR]), stable disease (SD), and non-responders for progressive disease (PD).

In this study, no statistical method was used to determine the sample size. The observed sample size provided a sufficient pool of both responders and non-responders, and produced performance measures (i.e., AUC, sensitivity, etc.) with adequate levels of precision. The study followed the Reporting Recommendations for Tumor Marker Prognostic Studies (REMARK). The study was approved by the Institutional Review Board. Sud Méditerranée V was obtained on June 26, 2018 (registration #2018-A02116-49; CNIL MR003 2221869 v0). This study was registered at ClinicalTrial.gov (NCT03584334).

**Blood samples**

Peripheral blood samples were collected in ethylenediaminetetraacetic acid-containing tubes before treatment. Truculture tubes (Rules Based Medicine, Austin, TX, USA) contained anti-CD3 and anti-CD28 antibodies to activate *in vitro* the T cell response in vitro by mimicking the first two activation signals [[2](#_ENREF_2)]. 1mL of whole blood was added to the thawed tube. The tube was incubated in a water bath at 37°C for 23 h and then stored at -20°C. The remaining volume of whole blood was centrifuged twice for 10 minutes at 2000 g and 4°C. The plasma obtained was aliquoted into cryovials and stored at -80 °C.

**Multiplex assay**

The Luminex® kit (#LXSAHM-24, R&D Systems, Minneapolis, MI, USA) was used to analyze a panel of 30 targets (IL1β, IL2, IL3, IL4, IL5, IL6, IL7, IL8, IL10, IL12p70, IL13, IL15, IL17A, IL18, CCL3, CCL4, CCL7, CCL17, CXCL1, CXCL2, CXCL5, CXCL6, CXCL9, CXCL10, CXCL13, IFNβ, IFNγ, MCP1, TNF-α, and PD-L1). The standard range was determined after reconstitution of Standard 1. A serial dilution of 1:3 was performed to obtain six standards. Diluent was used as a blank for the assay. 50μL μL samples/standards and 50μL μL microparticles were added to each well. Microparticles are magnetic beads, and each type of bead is covered with antibodies to capture the analyte of interest. Beads are distinguished based on their fluorescence properties. Incubation was carried out at room temperature on a plate stirrer (800 ± 50 rpm) after covering the plate with an aluminum film to prevent photobleaching of fluorochromes and evaporation of reagents. The plates were then incubated for 2 h. After incubation, the plate was fixed to a magnet to adhere the magnetic balls to the bottom of the wells. The wells were emptied by turning upside down and then washed three times with 100μL μL washing buffer.

After emptying the wells, 50μL of biotinylated antibody was added and the plate was incubated for 1 h. Three washes were performed, and 50μL of phycoerythrin (PE) coupled to streptavidin was added. The plate was incubated for 30 minutes before being washed 3 times. The microparticles were re-suspended in 100μL μL washing buffer. The plate was placed on an agitator for 2 min before being read using a Luminex® instrument. Each run was performed in duplicates. The duplicates did not vary by > 4%.

**ELISA assays**

Commercial sandwich ELISA kits were used to measure the plasma levels of TGF1β ((#DB100B, Quantikine ELISA Kit, R&D Systems), CCL17 (#DDN00, Quantikine ELISA Kit, R&D Systems), iNOS (#NBP2-80255, Bio-Techne, Minneapolis, MI, USA), and CXCL10 (#QK266, Quantikine ELISA Kit, R&D Systems), according to the manufacturer's instructions.

**Statistical analysis**

Welch’s test (normal conditions met) and Wilcoxon tests were used to compare the means of the two interest groups. The results of these tests were obtained after the Bonferroni correction. The generalized linear model (logistic regression) was used to generate different predictive models of responder status, and the selection of variables (30 targets) was carried out by correlation and then by the bottom-up, top-down, and manual methods. ROC curves and corresponding AUC were calculated to assess the effectiveness of the models. The significance threshold for the p-value was 0.05. Statistical analyses were performed using the R Studio 3.2.2.

**SUPPORTING RESULTS**

The study of the distribution of variables in the principal component analysis allowed the generation of a "manual" predictive model, two other models were generated by "machine learning" (logistic regression) via a bottom-up or top-down method (**Figure 3C**). With this data, we found a problem called "high dimensionality" because there were more variables than patients. Therefore, dimensionality reduction must be performed before selecting them for the model. Variables with a correlation coefficient higher than 0.8 were removed. Of the two correlated variables, we retained the one that provided us with the most information (lowest p-value), as follows: IL6, IL8, CCL7, IL10, MCP1, CXCL2, CXCL13, CCL4, IL1α, IL4 IL17A, IL13, IL5, IL12p70, IL1b, TNFα, and CD27.

The best-performing model was selected (top-down method; AUC=0.9532), and the number of variables was reduced to simplify it. This simplification of the model facilitated its extrapolation to other cohorts. The three combined variables with the highest predictive power were IL4, CXCL2, and TNFα (**Figure 3C**). High levels of IL4 were associated with an adverse response, whereas high levels of CXCL2 and TNFα were associated with a favorable response (**Figure 3C**).

**References**

1. Seymour L, Bogaerts J, Perrone A, Ford R, Schwartz LH, Mandrekar S, et al. iRECIST: guidelines for response criteria for use in trials testing immunotherapeutics. *Lancet Oncol*. 2017;18:e143-e52.

2. Duffy D, Rouilly V, Braudeau C, Corbiere V, Djebali R, Ungeheuer MN, et al. Standardized whole blood stimulation improves immunomonitoring of induced immune responses in multi-center study. *Clin Immunol*. 2017;183:325-35.
